# CDR-Net: A computerized framework to detect Alzheimer’s diseases and mild cognitive impairment

**DOI:** 10.1371/journal.pone.0346576

**Published:** 2026-04-20

**Authors:** Ashik Mostafa Alvi, Siuly Siuly, Maria Cristina De-Cola, Hua Wang

**Affiliations:** 1 Institute for Sustainable Industries and Liveable Cities, Victoria University, Melbourne, Victoria, Australia; 2 IRCCS Centro Neurolesi “Bonino-Pulejo”, Messina, Italy; University of Maryland at College Park, UNITED STATES OF AMERICA

## Abstract

Alzheimer’s disease (AD) and mild cognitive impairment (MCI) are two dementia-related brain illnesses that are prevalent among elders in the twenty-first century. MCI is treated as the initial stage of AD, and once the illness reaches the AD stage, there is no escape from certain death. The accuracy and efficacy of current multiclass computer-based approaches to diagnose AD and MCI are constrained by traditional machine learning (ML) classifiers due to their shallow architecture. This makes it challenging to make a prompt and accurate diagnosis of AD and MCI. This research proposes a framework employing electroencephalography (EEG) to diagnose MCI, AD, and healthy subjects (HSs) to boost multiclass performance and efficacy. EEG is a portable, non-invasive, and affordable means to identify brain problems as compared to expensive and time-consuming techniques like computed tomography (CT) scans, positron emission tomography (PET), magnetic resonance imaging (MRI), and the mini-mental state examination (MMSE). To circumvent these issues, the Cognitive Decline Recognition Network (CDR-Net) architecture has been developed to identify MCI, AD, and healthy individuals using EEG data. The proposed architecture allows for the acquisition of EEG data, data preprocessing (down-sampling, noise cleaning, segmentation, and digital picture construction), feature extraction and classification using CDR-Net, as well as performance assessment and cross-validation stages. Our suggested CDR-Net architecture produced better multiclass accuracy, sensitivity, and specificity of 99.25%, 99.13%, and 99.32%, respectively. By using 10 folds and leave-one-out cross validations, stability, consistency, and data overfitting and underfitting concerns are addressed. This framework will serve as a foundation for future systems designed to detect multiple brain disorders.

## Introduction

Mild cognitive impairment (MCI) and Alzheimer’s disease (AD) are both linked to dementias caused by neuronal damage. The death or malfunction of brain cells, called neurons, is a major contributor to these neurological disorders. Loss of memory, a reduced vocabulary, and a diminished capacity for precise motor motions are all signs of MCI and AD. Recent discoveries from studies indicate that patients with MCI have a significantly increased risk of developing dementia, and in particular AD [1–3]. MCI is regarded as the antecedent phase of AD [4]. With age, risk increases exponentially, with risk doubling approximately every 5–6 years and often affects those over the age of 65 [5–7]. The prevalence of MCI and AD is rising globally, with emerging nations expected to have a proportionally greater increase [8,9]. It ranks as Australia’s second-most common cause of death [10] and the seventh top death factor worldwide [11]. According to the 2018 World Alzheimer Report, 60 percent of dementia cases are attributable to AD. More than 50 million people worldwide are suffering from dementia. It is projected that there would be 152 million total cases by the year 2050, representing a more than threefold increase [12]. A patient with AD has a life expectancy of 5–8 years after being diagnosed, and MCI is also presently incurable [13,14]. Nonetheless, the diseases course may be slowed and the quality of life for patients can be enhanced by caretakers if the diagnosis is made early.

To diagnose MCI and AD, presently there are costly, invasive, and time-consuming methods available, including as magnetic resonance imaging (MRI), computed tomography scan (CT-scan), positron emission tomography (PET), and mini mental state evaluation (MMSE test) [15,16]. In contrast, electroencephalography (EEG) is a recently developed portable, affordable, simple to use and comprehend, and rapid technology to diagnose neurological illnesses including AD and MCI. The brain waves in the cerebral cortex relative to time is preserved by EEG recordings, which is the key to determining the severity of neurological illnesses [17]. The technique for collecting EEG data entails putting electrodes on the scalp in a specified pattern, with the international 10–20 approach being the most widely used configuration [18]. In light of this, electroencephalography’s potential as a tool for early diagnosis of MCI and AD has been emphasized.

Numerous investigations have been conducted over the past few decades in an effort to diagnose MCI at an early stage and prevent its progression to AD or another cognitive disorder. With 11 MCI and 4 AD participants, Morabito et al. [19] chose convolutional neural network (CNN) to accomplish a binary (AD vs. MCI) epoch-based classification. Power Spectral Density (PSD) was utilized to preprocess the acquired EEG data, and a multi-dimensional CNN with a softmax classifier model was then employed to conclude the binary classification. This attempt had an accuracy rate of up to 98.97% (95% confidence interval: 98.68%−99.26%). Two DL models—modified CNN and convolutional auto encoder (Conv-AE) neural networks (NNs)—were utilized in a recent study [20] to differentiate between 61 healthy subjects (HSs), 56 persons with MCI, and 63 people with AD. The EEG data were processed using time-frequency representation (TFR) and continuous wavelet transform (CWT) before being submitted to the NNs. Both the CNN and Conv-AE NN models had average accuracy of 92% and 89%, accordingly.

Ieracitano et al. [21] undertook an AD-MCI investigation using several machine learning (ML) techniques using 63 ADs, 63 MCIs, and 63 HSs. CWT and higher order statistics (HOS) from the bispectrum (BiS) features were retrieved and loaded into multi-layer perceptron (MLP), auto encoder (AE), logistic regression (LR), and support vector machines (SVM) classifiers. MLP fared better than the other ML classifiers in this investigation, surpassing them with an accuracy of 89.22%. Fast Fourier Transform (FFT) and Discrete Wavelet Transform (DWT) were implemented to acquire the spectrum characteristics and denoise the data in another traditional ML-based attempt [22] with 109 participants (49 ADs, 37 MCIs, and 23 HSs). The categorization challenge was handled by a Decision Tree (DT) using the C4.5 algorithm, which distinguished between HS and AD, HS and MCI, and MCI and AD with 83%, 92%, and 79% accuracy, correspondingly. A recent classical ML effort [23] was reported with 48 ADs, 37 MCIs, and 20 HSs, where investigators used PSD, finite impulse response (FIR), and 2nd order Butterworth filters for EEG data preprocessing. The classification task was carried out using the classifiers K-nearest neighbor (KNN), DT, and SVM. KNN outperformed the rest of the classifiers by gaining 97%, 95%, 83%, and 75% while performing HS vs. AD, HS vs. MCI, MCI vs. AD, and HS vs. MCI vs. AD classifications.

To provide a binary identification system utilizing a cubic-SVM, Puri et al. [24] studied 12 ADs and 11 HSs and reached a classification accuracy of 98.5%. The unprocessed EEG signals were divided into sub – bands applying low-complexity orthogonal wavelet filter banks with vanishing moments. The Kruskal-Wallis test was adopted to extract and examine the two characteristics, Higuchi’s fractal dimension (HFD) and Katz’s fractal dimension (KFD), from EEG sub-bands. A new research [25] employing MLP with 6 MCIs, 11 ADS, and 9 HSs was suggested and achieved an 88% F1 score. Using autoreject and independent component analysis (ICA) techniques, the investigators denoised the EEG data. The last step was to feed MLP with the extracted power, entropy, and complexity attributes. Poil et al. [26] presented a study with 86 subjects (25 ADs and 61 MCIs) to forecast the development of AD at the MCI stage. The authors extracted the features and passed them to the LR classifier for classification using the ICA and Hilbert Transform (HT). This binary categorization study achieved 88% sensitivity and 82% specificity. Kashefpoor et al. [27] collected primary EEG data of 16 HSs and 11 MCIs and conducted a study using Takagi–Sugeno neurofuzzy (NF) inference system along with KNN. This binary NF-KNN study reached 88.89% accuracy, 83.33% specificity, and 100% sensitivity.

Another binary classification research [28] based on deep learning with 28 ADs and 7 MCI participants was reported. Bidirectional long short-term memory (BLSTM) classifier was used after principal component analysis (PCA) was used to identify the features. With those aged 40–60, it had increased by 91.93%, and with those beyond 60, by 65.73% accuracy. Amezquita-Sanchez et al. [29] proposed an Enhanced Probabilistic Neural Network after studying 37 MCI and 37 AD participants (EPNN). ANOVA, Hurst Exponent (HE), Fractal Dimension (FD), and the MUSIC-Empirical Wavelet Transformation (EWT) were all investigated as ways to extract characteristics. To compare the classification outcomes, the DT, Naive Bayes (NB), and KNN were also applied. The EPNN outperformed the other used classifiers, with an accuracy of 90.3%. A limited sample size of four ADs, four HSs, and four MCIs was adopted by Bi and Wang [30]. The discriminative convolutional high-order Boltzmann machine (DCssCDBM) classifier received the features after being liberated by the FFT. 95.04 percent accuracy was achieved in this investigation.

From the reviewed articles, it is clear that none of the studies are able to break through multiclass AD-MCI detection with high accuracy and efficiency. Most of the reported efforts struggled to have a satisfactory sample size. Another significant finding is that effective research either identified standard ML approaches as a poor solution for multiclass issues or employed DL methods to address binary classification problems. And here comes one of the research problems that we intend to resolve is that the studies which performed better and used classical ML methods; they utilized feature extraction and selection methods for those ML classifiers. This extra step is always required when it comes to classical ML methods like SVM, LR, DT, KNN, and so on. Moreover, classical ML classifiers are limited and old-fashioned due to their shallow architecture and classical ML classifiers require manual feature extraction and selection and may underperform in multiclass scenarios compared to deep learning methods that automatically learn hierarchical features. One of our objectives is to reduce this computational expense by picking a suitable DL classifier that will not ask for any feature extraction or selection method. DL methods have passed the test of working with EEG data with flying colors [4,8,31–33]. Though, the performance of previous multiclass AD-MCI studies is really poor. Therefore, our goal is to set a high-performance standard for AD-MCI detection using EEG data.

In the past, we have explored long- and short-term memory (LSTM) [4], gated recurrent units (GRU) [8], and deep residual networks (DRN) [32] using EEG data to investigate for these sorts of brain illnesses. This study incorporates multiple well-known CNN models like AlexNet [34], InceptionNet [35], ResNet50 [36], and VGG16 [37]. After evaluating the performance and being inspired by the computational cost disadvantage, we are motivated to design a custom CNN model.

To address these problems, we offer the Cognitive Decline Recognition Network (CDR-Net) framework, which is a complete detection system package. It includes data recording, preprocessing, feature extraction and subject identification, and performance reporting phases which is why we refer it as a framework. Our proposed CDR-Net uses a specially created CNN model to conduct AD, MCI, and HS identification using EEG data. Both AD and MCI share the same symptoms of cognitive decline. Inspired by this, we named our framework the Cognitive Decline Recognition Network. It is well known that the CNN model works better with images [38], [39], so the EEG data we collect are processed and turned into images before being sent to the classifier. CNN is a compact classifier that uses less memory while processing pictures than other DL techniques [40]. Moreover, by itself, it is capable of extracting and choosing significant and in-depth characteristics. These considerations led to CNN being selected as the classifier for our CDR-Net framework. With the preprocessing steps, CNN parameters, and layers we have proposed, this is a whole new, inexpensive framework in terms of our novelty. An overview of this article’s achievements can be seen below:

- Developed and underpinned a modern, precise, reliable, and effective CDR-Net framework for detecting AD, MCI, and HS using EEG data.
- Achieved higher multi-class classification accuracy than previous methods across both identical and diverse EEG datasets.
- Examined the consistency and stability of this proposed CDR-Net by performing 10-fold and LOOCV cross validations.
- Conducted ablation tests to discover the best-suited CNN classifier, which is the heart of the CDR-Net framework.

The remaining sections of this study are arranged as follows: Section II talks about the proposed CDR-Net framework in detail. Afterwards, Section III reports on the experiments and findings. Following that, the discussion of this research is included in Section IV. Finally, Section V of this article provides a conclusion and a strategy for the future.

## Proposed CDR-NET framework

**Fig 1:**
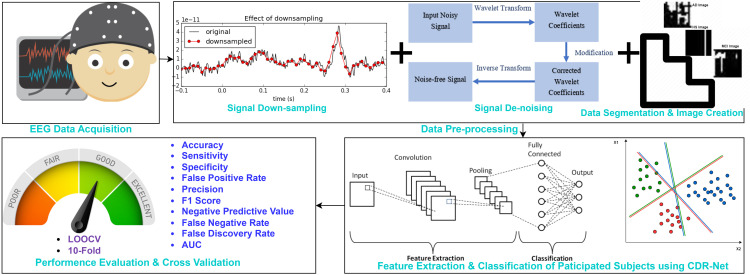
CDR-Net Framework.

This proposed CDR-Net framework has been built for multiple brain diseases detection using EEG data. Fig 1 showcases the proposed CDR-Net framework. The CDR-Net structure contains four phases, the first of which is the primary EEG data collecting phase. We were successful in gathering EEG data from 109 participants (23 HSs, 37 MCIs, and 49 ADs). Then, because there weren’t many EEG signals collected at a sampling frequency of 1024 Hz, the raw signals that were collected were down-sampled to 256 Hz to keep things consistent. To eradicate artifacts and noises from the raw signals, the stationary wavelet transform (SWT) has been performed. SWT is widely renowned for its ability to handle both high- and low-frequency noises. Afterwards, cleansed signals are segmented into 5-second frames in order to increase the sample size and acquire key information quickly. Before sending the temporal segmented frames to the classifier, they are finally converted to 8-bit color pictures. A specially created multi-layer CNN model with a softmax classifier completes the CDR-Net architecture. This custom-made CNN consists of multiple convolutional and max pooling layers for feature extractions. There is a fully connected layer for the classification purpose from the selected features. Finally, different performance measures have been used to evaluate this proposed CDR-Net system, which can identify multiple classes of cognitive abnormalities. To verify the consistency and stability of our presented CDR-Net architecture, the 10-fold and leave one out cross validations (LOOCV) were carried out. A comprehensive description of the four stages along with their sub steps are reported below:

### A. EEG Data Acquisition

The CDR-Net framework has been tested and trained with a multiclass EEG dataset holding 109 subjects’ EEG recordings: 23 HSs, 37 MCIs, and 49 AD subjects [23,22]. The local Ethics Committee of the IRCCS Centro Neurolesi “Bonino-Pulejo” approved the retrospective collection of data from the neuroimaging laboratory’s archive of samples (reference number: 40/2013). The data was extracted and pseudo-anonymized on 19 December 2014. Only the IRCCS author (i.e., MCDC) had access to information that could identify individual participants during or after the data was collected. All the participants had given their consent by signing the consent form.

This multiclass EEG data obtainment was performed using 19 electrodes (Fp1, F3, C3, P3, O1, F7, T3, T5, Fz, Cz, Pz, Fp2, F4, C4, P4, O2, F8, T4, T6) placed across the scalp pursuing international 10–20 system’s guideline [8]. All the subjects were advised to keep their eyes closed and remain rested until the data collection is done. Collected brain signals were evaluated with reference to electrical potentials (µV). Each recording lasted for approximately 5 minutes (300 seconds), keeping the sampling frequency at either 256 Hz or 1024 Hz. This uneven sampling frequency and duration have been fixed in the pre-processing step.

Following the Diagnostic and Statistical Manual of Mental Disorders (fifth edition, DSM-5) [23], all the participated subjects are classified as AD, MCI, or HS. Participants who were able to undergo an electroencephalogram and had no prior history of neurological Comorbidities were included in the study, whereas individuals receiving pharmacological treatments that could influence brain activity were excluded. Any subjects with a history of head trauma, drug abuse, serious medical conditions, or other forms of dementia were excluded from this study. The average ages of patients diagnosed with AD and MCI are 78.4 ± 6.4 and 74.1 ± 9.4 years, respectively, meanwhile the average age of HSs is 65.6 ± 7.9 years. Table 1 represents the demographic data of participated subjects.

**Table 1:** Demographic data of participated subjects.

Classes	Number of Subjects (%)			Mean age (SD) in years		
	Male	Female	Total	Male	Female	Total
MCI	17 (46%)	20 (54%)	37	75.7 ± 9.7	72.7 ± 9.1	74.1 ± 9.4
AD	20 (41%)	29 (59%)	49	78.6 ± 4.1	78.2 ± 7.6	78.4 ± 6.4
HS	13 (56%)	10 (44%)	23	68.1 ± 6.9	62.3 ± 8.3	65.6 ± 7.9
Total	50 (46%)	59 (54%)	109	74.9 ± 8.2	73.6 ± 9.9	74.2 ± 9.1

### B. Data Preprocessing

Collected raw signals were digitized and converted to MATLAB files and then prepared for further processing. There are 3 pre-processing techniques applied. Initially, the recorded raw signals are down-sampled to 256 Hz. Down-sampled signals were denoised using the SWT method. Cleaned signals are segmented to 5 second chunks for better learning and time saving. Lastly, temporal segments are converted to Joint Photographic Experts Group (JPG) images. An elaborated description of these pre-processing steps is reported below:

1. Signal Down-sampling

EEG data collection took place for multiple days, and this is one of the big EEG primary data having AD, MCI, and HSs. A total 109 subjects participated in this EEG study and 256 Hz and 1024 Hz sampling frequencies are used for collecting their brain potentials.

As there were different sensors used, the sampling frequency was not consistent. For some subjects, it was 1024 Hz and for others it was 256 Hz. To bring consistency, we have down-sampled the 1024 Hz sampling frequencies to 256 Hz using MATLAB. From the literature it can be said 256 Hz is the standard sampling frequency [20–22,32]. Now, all 109 subjects’ EEG recordings have the same sampling frequency, which is 256 Hz.

2. Signal De-noising

EEG data collection is often affected by some external or internal factors known as artifacts or noises which may corrupt the actual data. Eye blinks, muscle movement, electrode pops, breathing patterns, power supply fluxes, interference (50 Hz), outlier readings, and other electrical activities are some of the internal and external sources of noise. Actual signal gets contaminated with these and often mislead to a different conclusion if they are not removed from the actual signal [41,42]. Therefore, it is a must to discard the noise before further processing.

Our proposed CDR-Net employed SWT to dispel artifacts and other undesirable signals. SWT is suitable for trading with both low and high-frequency unwanted signals, keeping the flat response at the highest position of the passing signal. Previous efforts have used SWT, DWT, CWT, etc. frequency domain noise removing methods to clean the input signal. Apart from that, the most valuable frequency bands that carry important features are from 0.5 Hz to 32 Hz. [43].

An 8th order Symlet wavelet “*sym9*” filter has been chosen for exterminating DC components of the input signal (0–0.5 Hz) and the baseline electric drift in each of the electrode channels. On the other hand, high frequency artifacts (32–128 Hz) along with decomposition of stationary wavelets have been performed with 2nd order estimation for each of the 19 channels.

**Fig 2:**
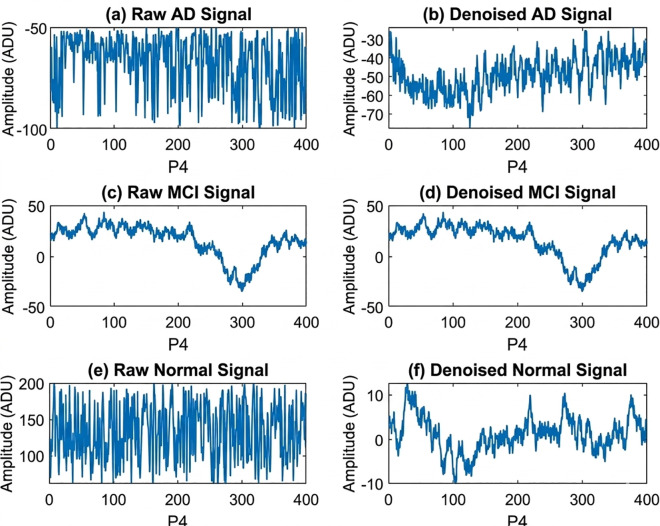
Visual EEG Data Cleaning Comparison of P4 Electrode Position. (a) raw EEG signal of an AD subject, (b) denoised signal of the same AD subject, (c) raw EEG signal of an MCI subject, (d) denoised signal of the same MCI subject, (e) raw EEG signal of a healthy subject, (f) denoised signal of the same healthy subject.

After the denoising phase, 109 cleaned signals are digitized representing electrical potentials (µV). Each signal has a length of 60 seconds × 5 minutes × 256 Hz and stored as a MATLAB (.mat) file. That gives us approximately 76800 rows representing voltage amplitude (µV) and 19 columns referring to 19 electrode channels for each subject. This phase of the proposed CDR-Net has been performed in MATLAB and a visual comparison of P4 electrode position of (a) raw EEG signal of an AD subject (3841–4240 seconds), (b) denoised signal of the same AD subject (3841–4240 seconds), (c) raw EEG signal of an MCI subject (21761–22160 seconds), (d) denoised signal of the same MCI subject (21761–22160 seconds), (e) raw EEG signal of a healthy subject (75521–75920 seconds), (f) denoised signal of the same healthy subject (75521–75920 seconds) are shown in Fig 2. It is a 400-second window, which has remained constant in all 6 sub-plots in Fig 2. Additionally, to assess the denoising performance, denoised signals are compared with the raw signal to measure the mean squared error (MSE) and peak signal-to-noise ratio (PSNR) in Table 2. Moreover, to justify the impact of denoising, same classification model has been utilized with raw and noised dataset and the results are visualized in Fig 5 – Fig 7.

3. Data Segmentation and Image Creation

**Table 2:** Quality measure after denoising.

Classes	PSNR		MSE	
	Male	Female	Male	Female
MCI	43.15	38.48	0.35	0.40
AD	40.33	33.04	0.39	0.42
HS	51.92	44.57	0.26	0.38

Segmentation is a technique which increases the sample size, reduces the computation expenses without losing any important feature or information. As shown in our earlier works, executing segmentation has a positive impact on the entire computing cost, which includes feature extraction, learning, model construction, classification, and testing [4,32]. It is well known that EEG data are bit different than electrocardiogram (ECG) or magnetoencephalography (MEG) data. EEG are complex, non-periodic, non-stationary and huge in terms of size compared to ECG or MEG. Moreover, managing enough medical data is a huge task. To address such a challenge, multiple studies [4,8,31,32] used data segmentation to increase sample size and deal with such complex EEG data.

In this proposed CDR-Net, EEG recordings are segmented into short time windows and tagged with the same label as the native signal. As noted in the section on EEG data obtainment, each recording lasted around 300 seconds. Investigations [4,8] on 10, 5, and 3 second segments show that 5 second segments are the best choice for keeping important features intact and allaying with time. If the segmentation duration is too large, it’s good in terms of keeping the features intact, but the computational cost raises with length. On the other hand, if the segmentation length is too low, the computational cost is very less, but the features are damaged. Our previous studies [4,8] showed that 5 or 6 seconds segments is the suitable option while balancing the feature set and time. In the context of this study, the approximate 300-second denoised recordings of each participant are cut up into non-overlapping uniform 5-second pieces.

Here is the math to get the new temporal segments number right: For AD1 subject, the recording ran for 745.28 seconds, giving us (745.28 seconds x 1024 Hz) = 763169 raw data rows. After down-sampling to 256 Hz, the same subject received (256 Hz x 745.28 seconds) = 190792 data rows. When, we segmented this AD1 subject into 5 seconds interval, it produced (190792/((256*5)) = 149 AD temporal segments. Initially (raw data), both the sampling frequency and recording duration were uneven for all subjects. Hence, not all subjects produced same number of temporal segments. Likewise, AD2 produced 153 AD temporal segments, AD3 produced 120 AD temporal segments; likewise, MCI1 produced 144 MCI temporal segments, HS1 produced 103 HS temporal segments.

The final tally of segments produced by this technique is 4525 for AD, 3789 for MCI, and 1663 for HS. Each newly created temporal segment includes 1280 rows (5 seconds × 256 Hz) and 19 columns (19 channels). Using the MATLAB’s *imwrite* function, all the temporal segments are converted to 8-bit color images (.JPG) for better feature picking by the CNN classifier. In summary, the sample size for this study has increased from 109 to 9977. Furthermore, these small temporal segment images help the classifier to select and extract important features easily.

### C. Feature extraction and classification of participated subjects using CDR-Net

**Fig 3:**
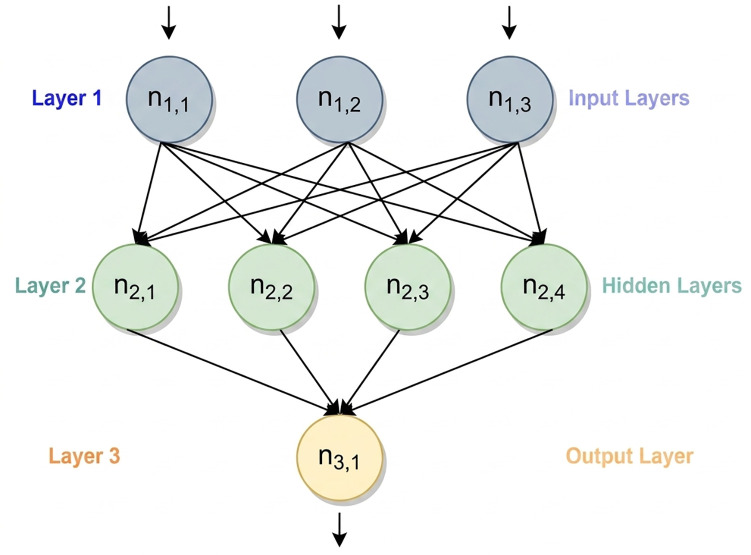
The structure of a classical ANN.

The fundamental objective of this multiclass work is to discriminate persons with MCI and AD from normal controls with an accuracy and efficiency that is sufficient by using non-stationary data such as EEG. Having said that, we have investigated multiple CNN models for this multiclass EEG dataset. CNN has grown its effective range from regular color or grayscale imaging to medical imaging like computed tomography images, x-ray images, spectrogram images, and magnetic resonance images. CNN with EEG data to identify brain diseases like MCI and AD are yet to be unraveled. CNN is a subtype of deep learning (DL), which is an enhanced form of artificial neural networks (ANNs). A classical ANN is shown in Fig 3, consisting of three layers: input, hidden, and output layers. Inspired by the structure of biological neurons in the human brain, an ANN is a collection of interconnected neurons, referred to as nodes in DL. The scaled total provided by the neurons in the preceding layer is applied to the output of the neurons. Similar to ANNs [44], the weights and biases of the prior layers in the network structure are utilized to determine the final output of the CNN model. Therefore, considering (1) and (2), the biases and weights are updated for each layer, where *W*, *B*, *u*, *L*, *s*, *C*, *m*, λ, and *t* denote the weight, bias, upda*t*ing step, learning rate, strata number, cost function, momentum, regularization parameter, and total number of training samples, respectively.


$$\Delta W_{s}(u+1)=-\frac{L}{r}W_{s}-\frac{L}{t}\frac{\partial c}{\partial W_{s}}+m\Delta W_{s}(u)$$ (1)

$$\Delta B_{s}(u+1)=-\frac{L}{t}\frac{\partial c}{\partial B_{s}}+m\Delta B_{s}(u)$$ (2)

CNN is made up of three different types of layers: Convolutional layers, pooling layers, and completely linked layers [45]. The feature extraction process makes use of convolutional and max-pooling layers. Max-pooling layers are intended for feature selection, while convolution layers are aimed for feature recognition [46]. When an image doesn’t need all the high-resolution information or when a reduced output with CNN-extracted areas is required after a down-sampling procedure on the input data, max-pooling layers are used [47]. The fully connected layers receive the outputs from the convolution and pooling layers and utilize them to categorize the input.

**Fig 4:**
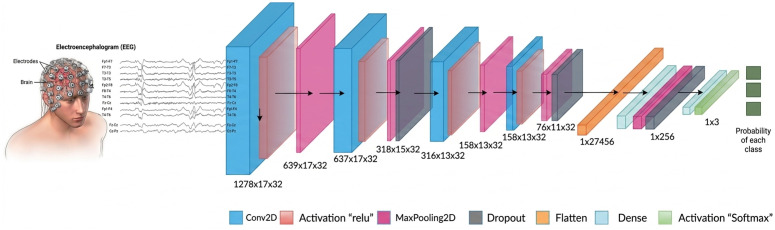
The proposed configuration of CNN classifier.

Our proposed CDR-Net employs a custom CNN model that consists of four feature extraction blocks. A convolution layer a *relu* activation function, and a max-pooling layer make up each feature extraction block. There are 32 filers in each convolution layer, and the kernel size is 3 × 3, while the pool size of max-pooling layer is 2 × 1. There are two 25% dropout layers present after the 2^nd^ and 4^th^ feature extraction blocks. Prior to sending the extracted features to the fully linked layer, there is a 50% dropout layer. Our custom-built CNN model incorporates the *softmax* classifier, the *adam* optimizer, and *categorical cross_entropy* as the loss function. Fig 4 depicts this custom-built CNN in a visual manner, along with the output shapes of each layer.

To make sure the classifier does not *overfit* and has the best model, we have brought early stopping into play. It has kept an eye on the validation loss and accuracy. The *min_delta* has been set to 0.001 and the *patience* to 15.

### D. Performance evaluation and cross validation

The fundamental objective of this multiclass work is to discriminate persons with MCI and AD from normal controls with an accuracy that is sufficient by using non-stationary data such as EEG. In light of this, we looked at more than twenty distinct CNN setups and reported the top five configurations and their performances in Table 7.

To make sure the best model gets reported, multiple performance matrices are in place. All of the explored setups are verified through (3), (4), (5), (6), and Receiver Operating Characteristic (ROC) graph. The ROC curve represents the relationship between the true positive rate (sensitivity) and the false positive rate (1 – specificity) on a graph. Often, the effectiveness of the classifier is shown by the ROC curve. The area under the ROC curve (AUC) is a statistical measure of how well the curve predicts the true outcome. The AUC value runs between 0 and 1. An AUC value around 1 and a standard deviation below 0 (not negative) indicate that the proposed method is performing optimally [48].


$$\text{Accuracy (ACC)}=\frac{TP+TN}{TP+TN+FP+FN}\times 100$$ (3)

$$\text{Sensitivity (SEN)}=\frac{TP}{TP+FN}\times 100$$ (4)

$$\text{Specificity (SPE)}=\frac{TN}{TN+FP}\times 100$$ (5)

$$\text{False Positive Rate (FPR)}=\frac{FP}{FP+TN}\times 100$$ (6)

Above, true positive (TP), true negative (TN), false positive (FP), and false negative (FN) are the four parameters that have been generated for each of the three classes.

A sample confusion matrix of how the TP, TN, FP, FN are calculated for class MCI is shown in Table 3. The green colored cell is representing TP (number of MCI samples correctly classified as MCI), the purple colored cells are standing for TN (number of non-MCI samples classified as either AD or HS), the light blue colored cells are referring to the FP value (number of AD or HS samples incorrectly classified as MCI), and the yellow colored cells are offering the FN value (number of MCI samples incorrectly classified as either AD or HS) for MCI class. The confusion matrices have multiple cells for TN, FP, and FN. Therefore, all the matching cells need to be summed together in order to calculate TN, FP, and FN. Similarly, for the AD and HS classes, the same calculations can be applied to obtain TP, TN, FP, and FN.

**Table 3:** Sample confusion matrices for three classes of classification while classifying MCI.

		Predicted Class			
Actual Class		HS	MCI	AD	
	**HS**	TP_HS_	*I*_HS,MCI_	*I*_HS,AD_	
	**MCI**	*I*_MCI,HS_	TP_MCI_	*I*_MCI,AD_	FN
	**AD**	*I*_AD,HS_	*I*_AD,MCI_	TP_AD_	TN
	${\;}^{*}I=\text{ Incorrect Classification }$		FP	TP	

Additionally, 10-fold and LOOCV are in place to test the performance consistency and stability of the proposed CDR-Net framework. Both approaches guarantee that the classifier is impartial and does not become too fitted to the data during the training process. While 10-fold cross validation is in progress, the entire preprocessed data are split into 10 chunks. During each fold, 9 split slices are utilized for training the model and one slice is kept for testing the model. Each of the data segments is examined once by repeating this method 10 times. The statistics of this 10-fold cross-validation confirm that the model is unbiased and consistent in terms of categorizing AD, MCI, and HS. Using the *KFold* method from the *sklearn* library, *shuffle* parameter has been turned true which makes sure the data are picked randomly, effectively grabbing samples for each fold using a uniform distribution across the entire EEG dataset. No additional manual measure has been taken to balance out the splitting so that same amount of data from each class belongs to each fold.

Lastly, LOOCV has been carried out to further guarantee the stability and absence of overfitting of the planned CDR-Net framework. As stated in the sub-section II-B-3, each of the subject’s EEG recordings is segmented into 5-second chunks and converted to 8-bit color images. Now, all the segments (color images) from a subject are left out from training the model and used for testing the trained model to predict the left-out subject [49]. All of the dataset’s individuals go through this procedure once. Despite being a computationally costly approach, it validates the stability, constancy, and impartiality of the suggested CDR-Net structure.

## Experiments and outcomes

The goal of this study is to develop an accurate model for detecting cognitive abnormalities from EEG data. For this process, CNN has been chosen because it is lightweight when it comes to images. This makes it possible for us to do the study in a quick manner. The setup and findings of the investigations are covered in this section.

### A. Experimental setup and tools

The CDR-Net structure is constructed and tested primarily using only two tools. Utilizing MATLAB R2021B, all preprocessing procedures are accomplished out, including signal down sampling, denoising, segmentation, and the production of 8-bit color images. The Python environment is used by Jupyter Notebook to carry out the feature extraction, classification, performance assessment, and cross validation phases. A Windows computer with 256 GB of RAM, an AMD Ryzen Threadripper PRO 3995WX 64-Core 2.70 GHz CPU, and an NVIDIA RTX A6000 graphics card was employed for all the investigations.

### B. Findings

Our proposed CDR-Net framework has proven its capability of distinguishing MCI, AD, and HS quite accurately and efficiently. In this part, we have reported class, fold, and batch size (BS) wise performance for each of the three classes. To examine the performance pattern and choose the optimal BS, several BSs were employed. For each of the three classes, the outcomes of four BSs—32, 64, 128, and 256—are presented and contrasted.

**Fig 5:**
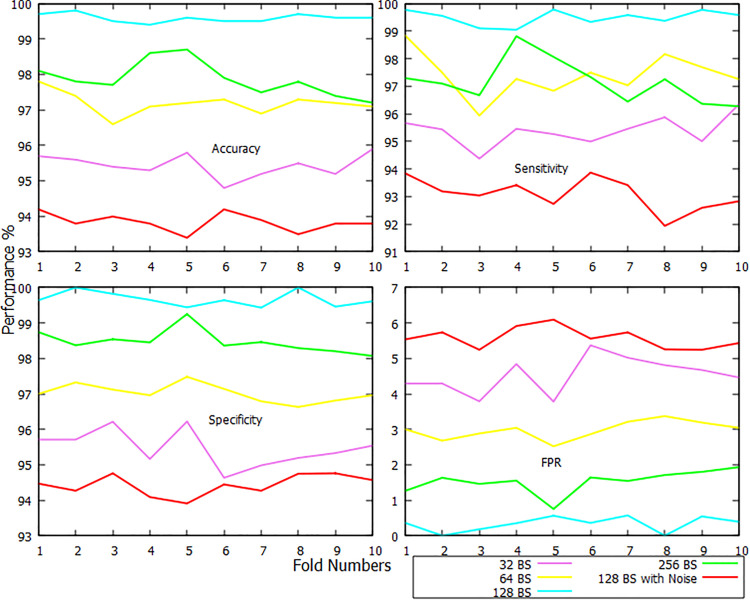
Fold-wise accuracy, sensitivity, specificity, and FPR visualization of HS class.

In Fig 5, the classification report of HS class has been visualized. This figure demonstrates that, when data overfitting and underfitting are taken into consideration, a BS of 128 is the optimal choice. Noised / raw dataset using the 128 BS has impacted the lowest performance while classifying HS subjects. It has received average 93.83% of accuracy, 93.08% of sensitivity, 94.43% of specificity, and 5.57% of FPR. The proposed CDR-Net has received the highest average accuracy of 99.59%, sensitivity of 99.49%, specificity of 99.67%, and FPR of 0.33% when the batch is set to 128. The second fold of BS 128 has performed better compared to the rest of the folds. The average accuracy, sensitivity, specificity, and FPR of 10 folds with a 32-BS are 95.43%, 95.38%, 95.47%, and 4.53%, respectively. When compared to the other folds of this kind, the 6th fold of BS 32 has had the worst performance. On the other hand, the 10-fold average accuracy, sensitivity, specificity, and FPR values of BS 64 are 97.18%, 97.39%, 97.02%, and 2.98%, respectively, and those of BS 256 are 97.87%, 97.14%, 98.47%, and 1.53%, respectively. The BSs of 64 and 256 almost achieved similar performance, but 256 performed better than 64 in terms of accuracy, specificity, and FPR. It can also be seen that BS 32 did not perform well compared to the other 3 BSs, and it is due to data underfitting. The sample size has been raised from 109 to 9977 since the preprocessing phase. And this action has enabled the classifier to capture features from a larger BS. However, due to the overfitting issue, the performance of the proposed CDR-Net starts to decrease when the BS goes beyond 128.

**Fig 6:**
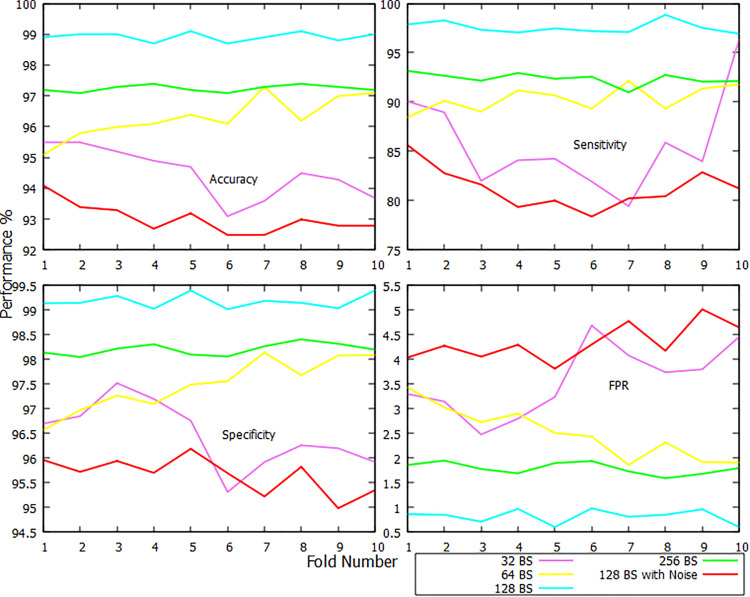
Fold-wise accuracy, sensitivity, specificity, and FPR visualization of MCI class.

The classification report for the MCI class has been illustrated in Fig 6. The CDR-Net has been successful in discriminating MCI from other samples 98.92% of the time on average when the BS is set to 128. Like Fig 5, a disastrous performance has been captured by the noised dataset in Fig 6 while differentiating MCI subjects. The average performance of CDR-Net while classifying MCI is: 98.92% accuracy, 97.58% sensitivity, 99.18% specificity, and 0.82% FPR, keeping the BS at 128. The maximum accuracy of this setup, over 99%, has been obtained by the 5th and 8th folds. The 10-fold average performance has decreased when the BS is kept at 32 and 64, giving an accuracy of 94.49% and 96.30%, a sensitivity of 84.45% and 90.28%, a specificity of 96.46% and 97.50%, and FPR of 3.54% and 2.50%. The proposed framework has improved the 10-fold average performance when the BS is increased to 256, giving accuracy of 97.24%, sensitivity of 92.42%, specificity of 98.21%, and FPR of 1.79%. The poorest 10-fold average performance has been reported when the BS is set to 128 with raw/noised dataset, returning accuracy of 93.02%, sensitivity of 81.25%, specificity of 95.66%, and FPR of 4.34%, with the 6th and 7th folds reporting the lowest. Again, BS 128 has won the race, outperforming the rest of the BSs by 10 folds on average.

**Fig 7:**
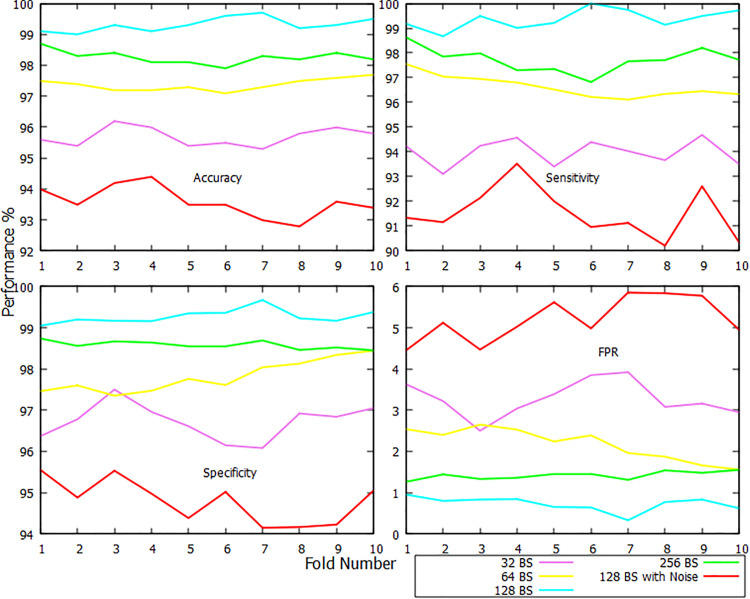
Fold-wise accuracy, sensitivity, specificity, and FPR visualization of AD class.

The classification report for the AD class is no different, delineating that BS 128 is the optimal one shown in Fig 7. The 10-fold average performance has again crossed over 99% when the BS is kept at 128. Such a BS setup produced an accuracy of 99.31%, sensitivity of 99.36%, specificity of 99.27%, and FPR of 0.73%. All the folds in this setup have reported over 99% accuracy, but the sixth has the highest. For this contest, the BS of 256 is likewise quite close, with reported averages of 98.26% accuracy after 10 iterations, 97.72% sensitivity, 98.58% specificity, and 1.42% FPR. The BS of 64 has come in third place, obtaining an average accuracy of 97.37%, a sensitivity of 96.63%, a specificity of 97.82%, and a FPR of 2.18% across a 10-fold range. A 32-BS has an average 10-fold accuracy, sensitivity, specificity, and FPR of 95.69%, 93.99%, 96.72%, and 3.28%, respectively, with the most accuracy being contributed by the sixth fold and the lowest by the seventh. Lastly, with 128 BS and raw dataset has stood last one more time, claiming 93.58% of accuracy, 91.54% of sensitivity, 94.79% of specificity, and 5.21% of FPR. Again, it is a clear win for CDR-Net when the BS is set to 128.

The overall multiclass classification debriefing reported in Table 4 tells us about the consistency and high classification performance of our proposed CDR-Net framework. The excellent detection accuracy illustrates how well CDR-Net can cope with a variety of brain illnesses. In addition, Table 4 showcases fold-wise multiclass performance. For BSs of 32, 64, 128, and 256, the average multiclass accuracy is 95.21 percent, 97.0 percent, 99.25 percent, and 97.87 percent, respectively. The CDR-Net framework’s average multiclass sensitivity for BSs of 32, 64, 128, and 128 is 94.98%, 97.16%, 99.13%, and 97.17%, correspondingly. Additionally, the average multiclass specificities for BSs of 32, 64, 128, and 256 are 95.39%, 96.99%, 99.32%, and 98.47%, accordingly. With the 128 BS and preprocessed EEG data (except the image conversion stage) has also been in action to determine the impact of medical imaging in neuro disorder detection. It has gained average 96.96% of accuracy, 96.56% of sensitivity, and 97.31% of specificity. The standard deviation (SD) row shows the variability of each metric across the 10 folds, where lower SD values—particularly in the 128 batch size configuration—indicate more stable and consistent model performance. In contrast, the confidence interval (CI) row reflects the precision of the estimated mean performance, with narrower confidence intervals demonstrating higher reliability of the reported averages. Together, these rows highlight that the 128-batch size (with image conversion) provides the most consistent and statistically precise results among all configurations. BS 256 outperforms BS 64 and BS 128 EEG version in terms of performance.

**Table 4:** OVERALL MULTICLASS CLASSIFICATION REPORT.

BS	32			64			128			256			128 (without image conversion–EEG version)		
Fold No	ACC	SEN	SPE	ACC	SEN	SPE	ACC	SEN	SPE	ACC	SEN	SPE	ACC	SEN	SPE
1	95.09	94.97	95.19	97.39	98.37	96.66	99.20	99.18	99.21	98.00	97.29	96.61	98.56	97.19	97.67
2	94.89	94.75	95.00	96.49	96.11	96.79	99.20	98.94	99.36	98.10	97.52	96.72	98.56	97.09	97.47
3	95.19	93.92	96.21	96.79	96.15	97.31	99.50	99.49	99.50	97.70	96.67	96.73	98.54	96.18	96.75
4	95.29	95.25	95.32	97.29	97.48	97.15	99.30	99.26	99.33	98.00	97.85	96.80	98.10	97.49	97.85
5	95.59	95.24	95.87	97.19	96.83	97.48	99.20	98.94	99.36	97.70	97.19	96.88	98.13	97.10	97.65
6	94.39	94.33	94.43	96.99	96.82	97.13	99.30	99.20	99.36	98.00	97.33	96.81	98.05	96.41	97.28
7	94.89	94.58	95.14	97.09	97.25	96.77	99.00	99.74	99.84	98.00	96.65	96.85	98.05	96.59	97.51
8	95.09	94.98	95.17	97.19	97.93	96.62	99.10	98.57	99.38	98.09	97.46	96.84	98.06	96.41	97.33
9	95.59	95.45	95.69	96.99	97.25	96.79	99.00	98.99	99.00	97.79	96.61	96.81	98.74	96.59	97.14
10	96.09	96.36	95.88	97.19	97.48	96.97	99.08	98.87	98.91	97.49	96.88	96.86	98.06	96.23	97.62
AVG	**95.21**	**94.98**	**95.39**	**97.06**	**97.16**	**96.99**	**99.25**	**99.13**	**99.32**	**97.87**	**97.14**	**96.80**	**98.47**	**96.56**	**97.31**
SD	0.46	0.58	0.36	0.26	0.58	0.37	0.11	0.33	0.21	0.21	0.36	0.19	0.19	0.47	0.31
CI (mean)	95.21	94.98	95.39	97.06	97.16	96.99	99.25	99.13	99.32	97.87	97.14	96.8	98.47	96.56	97.31

In this study, we have not only considered the accuracy of our proposed CDR-Net framework but also its efficiency. Table 5 showcases the efficiency of each fold and different BSs. In comparison to the other BSs, the 32 BS has needed the fewest average training epochs (24.9), on average. On the contrary, the BS of 64 spent the most time training the model, averaging 29.6 epochs. The BSs of 128 and 265 have consumed 27.4 and 26.5 epochs on average, respectively. Lastly, BS 128 EEG version has claimed the highest number of epochs, 33.6 on average, adding additional time to train. This 128 BS EEG version has consumed on average 48.69 seconds per epoch and become the most time costly model that we have tested. Again, according to Table 5, the shortest average time per epoch for a BS of 128 is 2.46 seconds. The BSs of 32, 64, and 256 have recorded averages of 3.16, 3.2, and 2.76 seconds per epoch, respectively. These evaluations show that training with a BS of 128 has taken less time overall for each fold. Despite the fact that this configuration has taken more than 27 epochs on average in each fold, which is the third lowest of the four BSs, it is well ahead of its league. Additionally, the 128 BS EEG version has proven that the image conversion has made a huge impact on the execution and training time.

**Table 5:** FOLD AND BS WISE TIME COMPLEXITY REPORT.

BS	32		64		128		256		128 (without image conversion–EEG version)	
Fold No	#Epochs	Avg time per epoch (seconds)	#Epochs	Avg time per epoch (seconds)	#Epochs	Avg time per epoch (seconds)	#Epochs	Avg time per epoch (seconds)	#Epochs	Avg time per epoch (seconds)
1	27	3.22	30	3.28	33	2.84	22	2.98	36	49.78
2	30	3.14	28	3.18	36	1.97	29	2.66	37	49.97
3	28	3.14	40	3.18	24	2.47	18	2.73	31	46.66
4	20	3.16	25	3.18	25	2.50	41	2.68	32	48.87
5	19	3.16	31	3.18	29	2.37	28	2.69	34	50.01
6	33	3.14	33	3.18	36	2.43	31	2.72	43	49.51
7	32	3.14	29	3.22	26	2.53	25	2.81	32	47.23
8	17	3.17	31	3.21	27	2.53	18	2.61	32	47.33
9	21	3.16	25	3.18	20	2.59	38	2.76	30	49.10
10	22	3.15	24	3.22	18	2.57	20	2.96	29	48.43
**AVG**	**24.9**	**3.158**	**29.6**	**3.201**	**27.4**	**2.464**	**26.5**	**2.76**	**33.6**	**48.689**

Table 6 presents a rigorous statistical comparison between the best-performing model (BS = 128) and all other configurations. Both parametric (paired t-test) and non-parametric (Wilcoxon signed-rank test) analyses confirm that the BS = 128 model is statistically superior. The paired observations are the 10-fold cross-validation accuracy values, where each fold’s accuracy for the 128-BS model is directly paired with the corresponding fold’s accuracy from the comparison model (n = 10 paired observations, degrees of freedom-df = 9). The extremely low p-values from the paired t-tests (all ≤ 5.71 × 10^−8^) provide overwhelming evidence of significant performance differences. The Wilcoxon p-values, while larger at 0.001953, remain well below the conventional 0.05 significance threshold, reinforcing the conclusion that the differences are not due to random chance. The comparison with the 128 (EEG) version is particularly revealing, showing that even with the same batch size, the image conversion method provides a statistically significant advantage in accuracy. These results validate BS = 128 as the optimal configuration with a high degree of statistical confidence.

**Table 6:** Statistical Comparison of All Batch Sizes vs. Best Model (128 BS) Using 10-Fold Accuracy Values.

Model Compared To	Paired t-test p-value	Wilcoxon p-value
32-ACC	1.10 × 10^−9^	0.001953
64-ACC	6.04 × 10^−9^	0.001953
256-ACC	2.16 × 10^−8^	0.001953
128-without image conversion–EEG version-ACC	5.71 × 10^−8^	0.001953

**Fig 8:**
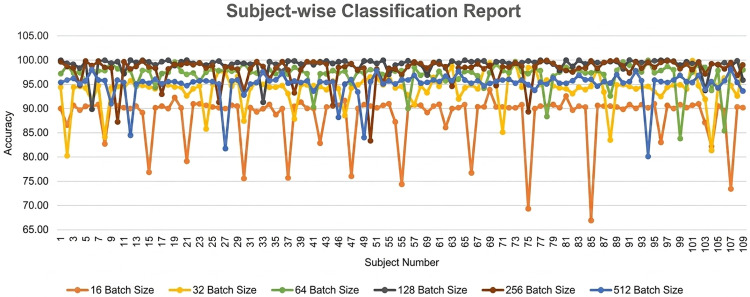
Subject-wise Classification Report.

To validate the stability and absence of overfitting, LOOCV has been performed. All segments of a subject have been kept aside from training and used for testing to predict the subject’s status. This strategy continues until all the subjects are checked. With the use of LOOCV, we have conducted subject-wise detection on the main EEG data that are obtained from 109 participants in this study. Along with the earlier 32, 64, 128, and 256 BSs, we have also documented the performance of BSs 16 and 512 in this LOOCV phase. The average accuracy for batches of 16, 32, 64, 128, 256, and 512 is 88.61 percent, 94.1 percent, 97.0 percent, 99.10 percent, 98.0 percent, and 95.0 percent, respectively. Different BSs and subject-wise accuracy are visualized in Fig 8, where the X axis represents the subject numbers, and the Y axis represents the corresponding accuracy. The 16, 32, 64, 128, 256, and 512 subject-wise accuracy of BSs are shown in Fig 8 by the orange, yellow, green, black, brown, and blue lines respectively. Among different BSs, 128 and 256 have performed better when LOOCV is in action. The worst performance has been reported for BS 16 due to data underfitting.

In order to strengthen the suggested CDR-Net framework, several performance matrices, 10-fold, and LOOCV are being used. It is abundantly evident from both 10-fold and LOOCV results that a BS of 128 is the best option for this dataset. Apart from that, over twenty CNN configurations have been investigated, and the top five setups are listed in Table 7 before recommending the best CNN configuration for our CDR-Net architecture. In the context of ML, and particularly sophisticated deep neural networks, a technique where certain components of the network are deleted or added in order to better understand the behavior of the network has been referred to as “ablation studies.” Table 7 compiles the results of the ablation investigation performance while maintaining a BS of 128. Here are the top five configurations along with well-established and popular CNN models depicted in Table 7, where a feature extraction block has been inserted and eliminated, the filters in the convolution layers are cut in half and doubled, and then the kernel window size has been raised to 5×5. All these configurations’ 10-fold and LOOCV performances indicate that the proposed baseline CNN configuration is the optimal one. The classifier’s performance suffered significantly when a feature extraction block being added or removed, but less so when the filter or kernel size has been altered.

**Table 7:** ABLATION STUDY.

Configurations	AVG Accuracy_10_Folds	AVG Subject-wise Accuracy LOOCV
**Proposed baseline setup**	**99.25**	**99.09**
Added a feature extraction block	96.87	98.49
Removed a featured extraction block	94.60	93.03
Halved the filters in the convolution layers	98.19	97.54
Doubled the filters in the convolution layers	98.78	98.10
Changed the kernel size to 5 × 5	98.53	97.47
AlexNet [34]	74.38	69.44
InceptionNet [35]	81.92	76.63
ResNet50 [36]	94.35	89.86
VGG16 [37]	89.47	87.30

Moreover, when trained on our EEG multiclass dataset, the popular CNN models AlexNet [34], InceptionNet [35], ResNet50 [36], and VGG16 [37] failed to provide satisfactory results. Additionally, since they are complicated layered CNN models, extra time was spent on training and testing. Only ResNet50 has achieved an average 10 –fold and LOOCV multiclass accuracy of 94.35% and 89.86% respectively and, with the other CNN models lagging behind at 90%. The possible reasons behind this poor performance from the established CNN models could be their kernel size, the number of max-pooling and convolutional layers and the activation function. Hence, the suggested design has been justified by the ablation work

**Fig 9:**
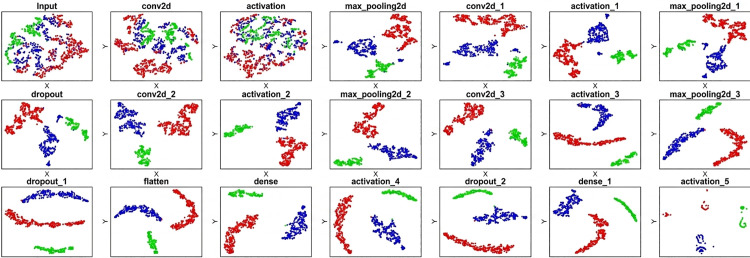
T-SNE Visualization of the Categorization Process.

Since the feature extraction process is so intricate and hard to comprehend, DL is sometimes referred to as a “black box.” T-distributed stochastic neighbor embedding (t-SNE) [50] is a widely used dimension reduction method which allows to visualize high dimensional like EEG by mapping it to a 2-D space. DL investigators often use t-SNE to visualize the categorization process. In Fig 9, the layer-by-layer visualization of the second fold of the proposed CNN configuration’s testing phase with a BS of 128 model is illustrated. This image shows that all of the samples are initially clustered together in the input layer for this testing, and that as it advances through the feature extraction blocks, the testing samples become dispersed. The proposed four feature extraction blocks make sure all the AD, MCI, and HS samples are clustered away from each other to have an accurate model. The testing samples are all clearly segregated from one another by the time they reach the dense layer.

**Fig 10:**
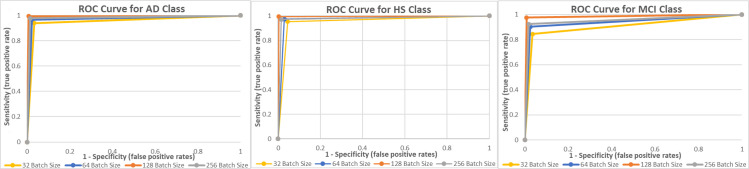
ROC Curves. (a) AD Class, (b) HS Class, and (c) MCI Class.

Moreover, the class-wise ROC curves are showcased in Fig 10. The ROC curves in yellow, blue, orange, and grey represent BSs of 32, 64, 128, and 256. The orange ROC curve, which reflects the 128-BS, has a substantially larger area underneath it in all 3 classes. This indicates a higher AUC reading. The ROC curve and the AUC value both complement one another and elevate the classifier.

## Discussion

This CDR-Net system has been developed to diagnose AD, MCI, and HS using EEG data swiftly and correctly. To do so, we have accumulated the EEG data of 109 people, 49 of whom have ADs, 37 have MCIs, and 23 have HSs. In order to clean the data and increase the sample size, the raw EEG data was down-sampled, de-noised by SWT, segmented, and transformed into an 8-bit color image before being sent to the CNN model. The CNN model has been tested in several setups, and Table 7 reports the results. Our proposed CDR-Net structure has a 99.25%, 99.13%, and 99.32% average multiclass classification accuracy, sensitivity, and specificity. 10-folds, LOOCV, and other performance matrices have been employed to demonstrate the performance.

Performance issues and computational complexity impeded earlier efforts to diagnose MCI or AD. The majority of the studied literature used traditional ML techniques to successfully conduct binary classification. However, the performance drastically deteriorates when trying to identify various brain illnesses. It is caused on by data noise, a limited sample size, the drawbacks of traditional ML techniques, and, last but not least, the absence of crucial features. MCI and AD share some similar symptoms. The majority of earlier multiclass initiatives misclassified MCI as AD since they share several symptoms. Moreover, traditional ML classifiers also need distinct techniques for choosing features and identifying them. Such two additional processes often introduce errors or leave out key features, and they have some computing expenses associated with them. Since they are unable to extract features on their own, the traditional ML classifiers rely heavily on these feature extraction techniques.

Table 8 provides a comparative summary of our proposed CDR-Net architecture, earlier investigations, and other well established CNN models. The majority of earlier work used the conventional ML classifiers SVM, DT, KNN, MLP, and LR [21–27,29]. For the preprocessing stage, the bulk of ML-based efforts used CWT, FFT, PSD, ICA, and PCA algorithms. With DL classifiers like CNN, BLSTM, DCssCDBM, and EPNN, four investigations were included [19,20,28–30,32]. In addition, the majority of the investigations that are reported on employed binary classification and have an average accuracy rate of above 90%. Furthermore, only half of the twelve recently published relevant studies employed multiclass categorization. Our prior work [32] among them had the greatest multiclass classification accuracy, with a score of 96.26%, utilizing identical EEG dataset. The second-best multiclass model was constructed by Bi and Wang [30] for predicting MCI, AD, and HS. With a multiclass accuracy of 75% and the KNN as the classifier, Pirrone et al.’s [23] model has been found to be the least accurate for multiclass performance. This is due to the classifiers’ usage having a simplistic design, which resulted in improper feature extraction. Another important finding is the difficulty [24,25], and [30] have had in obtaining a sufficient sample size. A classical ML study [27] collected primary data and proposed NF-KNN based detection system which reached 88.89%. To validate the performance of our proposed CDR-Net framework, we have tested it with the [27] study’s dataset. Yet again, our CDR-Net has proved that it is not only suitable for our dataset, but also can be used with other EEG dataset as well.

**Table 8:** Comparison with Earlier Efforts.

Efforts	Dataset	Method	Classifier	Classes	Performance
Fouladi et al. [20]	61 HS, 56 MCI, and 63 AD	TFR, CWT	CNN, Conv-AE neural networks	HS vs MCI vs AD	CNN 92%, Conv-AE 89%
Ieracitano et al. [21]	63 AD, 63 HS, and 63 MCI	CWT, HOS, BiS	MLP, AE, LR, SVM	HS vs MCI vs AD	89.22%
Fiscon et al. [22]	23 HS, 49 AD, and 37 MCI	Discrete Fourier Transforms, DWT	DT	HS vs AD, HS vs MCI, and MCI vs AD	83%, 92%, and 79% respectively
Pirrone et al. [23]	48 AD, 37 MCI, 20 HS	PSD, FIR & Butterworth filter	KNN, DT, SVM	HS vs AD, HS vs MCI, MCI vs AD, HS vs MCI vs AD	97%, 95%, 83%, and 75% respectively for KNN
Perez-Valero et al. al. [25]	6 MCIs, 11 ADS, and 9 HSs	Autoreject, ICA	MLP	HS vs MCI vs AD	88% F1 score
Puri et al. [24]	12 ADs and 11 HSs	HFD, KFD	SVM	AD vs HSs	accuracy of 98.5%
Sridhar and Manian [28]	28 ADs and 7 MCI participants	PCA	BLSTM	AD vs MCI	With those aged 40–60, it had increased by 91.93%, and with those beyond 60, by 65.73% accuracy
Amezquita-Sanchez et al. [29]	37 MCI and 37 AD	ANOVA, HE, FD, and MUSIC-EWT	EPNN, DT, NB, and KNN	AD vs MCI	accuracy of 90.3% by EPNN
Bi and Wang [30]	four ADs, four HSs, and four MCIs	FFT	DCssCDBM	HS vs MCI vs AD	95.04 percent accuracy
Poil et al. [26]	25 ADs and 61 MCIs	ICA, HT	LR	AD vs MCI	88% sensitivity and 82% specificity
Kashefpoor et al. [27]	16 HSs and 11 MCIs	correlation-based pursuit	NF-KNN	HS vs MCI	88.89% accuracy
DRAM-Net [32]	Our Dataset	SWT	deep residual network	HS vs MCI vs AD	Accuracy 96.26%
Our Proposed CDR-Net Framework	Kashefpoor et al. [27] Dataset	SWT	CNN	HS vs MCI	Accuracy 99.53%
Our Proposed CDR-Net Framework	Our Dataset	SWT	CNN	HS vs MCI vs AD	Accuracy 99.25%

While traditional ML methods can achieve strong performance in binary classification tasks, their reliance on manual feature extraction and their relatively shallow architectures may limit their effectiveness in more complex multiclass scenarios compared to deep learning approaches. It is challenging for such techniques to penetrate deeply into intricate layers and retrieve the vital characteristic of EEG data. However, since these approaches need additional processing for feature extraction, they have large computational costs. These are the driving forces behind our study’s decision to use a DL classifier that does not need additional feature extraction techniques and reduces processing expenses. Additionally, the methodologies used by NNs make it possible to extract important elements from very complicated and deep layers of data. Specifically, CNNs are often employed in digital image processing research and save a significant amount of time during training and testing. Since the deep layers of the NNs are all linked, feature extraction does not need human processing. Our classification performance makes it clear that the proper decisions we have taken allowed us to achieve less than 1% FPR and over 99% accuracy. Consequently, this high performance is an indication of a good feature extraction and classification process. With an average of 27.4 epochs and 2.46 seconds per epoch, our suggested CDR-Net trained and tested the model for each fold in 67.404 seconds. It is a pointer to the effectiveness of our proposed framework.

This study focuses on broadband signal, not any specific frequencies. It would have been better to cross validate other external EEG dataset with our proposed framework. Additionally, increasing the sample size could eradicate the error rate more. Comparatively to existing methods, CDR-Net is a simple and highly accurate framework for classifying AD, MCI, and HS using EEG data.

## Conclusion and future work

The designed CDR-Net system presented here is particularly efficient and accurate in recognizing patients with AD and MCI from EEG data. The emphasis in this study is on decreasing diagnosis time, improving performance over existing efforts, increasing trust in EEG data, and selecting the best classifier to reduce the number of false positives and negatives. Since the initial EEG data we have gathered includes artifacts and is inconsistent, the preprocessing step is given more attention. The raw signals are cleaned using SWT, which has taken care of both high- and low-frequency noises. By down-sampling the data to 256 Hz, the unevenness has been addressed. For more effective feature extraction, data were divided into 5-second chunks and converted to 8-bit colored pictures at the completion of the preprocessing stage. Before settling on the CNN design with the maximum accuracy, we investigated into over twenty different CNN setups. The suggested CNN model has just taken over a minute to complete the training and testing process for each round. In order to verify our suggested framework, the performance of the CDR-Net architecture has been evaluated using 10-folds, subject-wise detection, and other considerable performance matrices. The final results show that the overall multiclass accuracy, sensitivity, and specificity for CDR-Net are 99.25%, 99.13%, and 99.32%.

Future investigations should concentrate on expanding the sample size and multiclass performance. A smaller sample size has an impact on the classifier’s performance. The more data we supply to the classifier, the more it can learn and predict correctly.Though JPG is a lossy format, but PSNR/MSE results demonstrate good signal quality after denoising. Future work can explicitly examine how much of the EEG’s spectral data is preserved through the image conversion process (e.g., via reconstruction or spectral comparison). In future work, it would be valuable to explore whether similar frameworks, potentially adapted or extended, could be applied to other brain disorders such as autism, schizophrenia, or Parkinson’s disease. We intend to develop a web-based technology that can identify many brain abnormalities using EEG data.
